# Multi-spectral Metasurface for Different Functional Control of Reflection Waves

**DOI:** 10.1038/srep23291

**Published:** 2016-03-22

**Authors:** Cheng Huang, Wenbo Pan, Xiaoliang Ma, Xiangang Luo

**Affiliations:** 1 State Key Laboratory of Optical Technologies on Nano-Fabrication and Micro-Engineering, Institute of Optics and Electronics, Chinese Academy of Science, P. O. Box 350, Chengdu 610209, China

## Abstract

Metasurface have recently generated much interest due to its strong manipulation of electromagnetic wave and its easy fabrication compared to bulky metamaterial. Here, we propose the design of a multi-spectral metasurface that can achieve beam deflection and broadband diffusion simultaneously at two different frequency bands. The metasurface is composed of two-layered metallic patterns backed by a metallic ground plane. The top-layer metasurface utilizes the cross-line structures with two different dimensions for producing 0 and π reflection phase response, while the bottom-layer metasurface is realized by a topological morphing of the I-shaped patterns for creating the gradient phase distribution. The whole metasurface is demonstrated to independently control the reflected waves to realize different functions at two bands when illuminated by a normal linear-polarized wave. Both simulation and experimental results show that the beam deflection is achieved at K-band with broadband diffusion at X-Ku band.

In the last decades, metameterial, as an artificially structured material, has attracted much attention and brought in many intriguing applications across the electromagnetic spectrum, such as negative refraction^1^,^2^, invisible cloak^3^,^4^, Fano resonance^5^ and super-diffraction imaging^6^,^7^. The metamaterial is generally classified into two types which are periodic structures and non-periodic structures based on its structure composition. The initial research focused on the bulky metamaterial with subwavelengh periodic structures. Through artificial design of meta-atoms, the arbitrary effective electromagnetic parameter not readily available in natural materials, including zero index^8^, negative index^1^,^2^ and high index^9^, can be constructed from this type of the homegeneous metamaterial, followed by many unique electromagnetic effects or phenomena^10^,^11^. Afterwards, with the further advance of the metamaterial, especially for the development of transformation optics^12^,^13^, the subwavelength non-periodic structures have been largely employed to design the inhomegeneous metamaterials. Compared with the homegeneous metamaterials, they have more design freedom, which are also more flexible to manipulate electromagnetic (EM) wave. In addition, with the aid of transformation optics and conventional geometrical optics method, many novel EM function devices have been created, such as invisible cloak^3^,^4^, flat lens^14^, and beam manipulation^15^,^16^. Nevertheless, metamaterial has some insuperable difficulties in manufacture and material loss, preventing its widespread application.

Recently, the introduction of metasurface significantly relaxes the fabrication requirement due to its small characteristic thickness much smaller than the wavelength. The propagation direction of electromagnetic waves could be altered by using the metasurface-assisted law of refraction and reflection (MLRR), opening a new era of wave manipulation^17^,^18^,^19^,^20^. Metasurface can be classified into transmission and reflection operation modes. For the transmission operation mode, several challenges impede large-scale use and further development of the ultra-thin metasurface. One of them is the intrinsically low transmission efficiency, defined as the ratio of the energy of the anomalous cross-polarized beam to that of the total incident wave. The theoretical upper limit for the transmission efficiency of the ultra-thin metasurface is demonstrated to be 25%^21^. In ref. 22, the Pancharatnam-Berry (P-B) phase elements were adopted to construct the transmission-type ultra-thin metasurface, and its transmission efficiency of the anomalous cross-polarized component reaches 24.7%, approaching the theoretical limit. If the thickness of metasurface is designed to be finite thin, higher transmission efficiency can be obtained^23^,^24^. Compared with the low transmission efficiency of the transmission-type metasurface, the metasurface operating in reflection mode has very high efficiency almost close to 100% to handle the reflection of the incident wave, as it can make the incoming wave totally reflected and meanwhile modulate the reflected wave by suitably tailoring the phase of each meta-atom. Through special arrangement of the meta-atoms in a space/size/shape-variant manner, the arbitrary phase distribution could be obtained^25^,^26^. So far, reflection-type metasurfaces based on the phase discontinuities have been realized in visible^17^, terahertz^18^ and microwave range^25^,^26^. In addition, the phase-manipulating metasurfaces have been also developed to many novel and intriguing applications, such as wave-front control engineering^27^,^28^, holography^29^, flat metalenses^30^, polarization converter^31^,^32^, low radar cross section (RCS)^25^,^33^,^34^,^35^ and so on. Compared with the RCS-reduction method based on absorption, this new approach does not rely on the resistance and environment, presenting many new promising apsects^25^,^35^. It is worth noting that most of the metasurfaces demonstrated so far, can achieve only one function or operate in a single frequency band. There is little work focusing on the design of multi-functional, multi-spectral metasurface.

In this article, we present a two-layered reflection-type metasurface in which two kinds of meta-atoms are adopted to control the phase at different frequencies. By appropriately designing meta-atoms on each layer, the metasurface can independently manipulate the reflected field distribution at each frequency as desired. Here, the top-layer metasurface is composed of the cross-line structures with two different dimensions for producing 0 and π phase response, while a topological morphing of the I-shaped patterns with gradient phase distribution are adopted to construct the bottom-layer metasurface. Both numerical simulations and experiments demonstrate that the designed metasurface can simultaneously achieve beam deflection at K-band and broadband diffusion at X-Ku bands.

## Results

### Structure design and its simulation results

**Figure 1:**
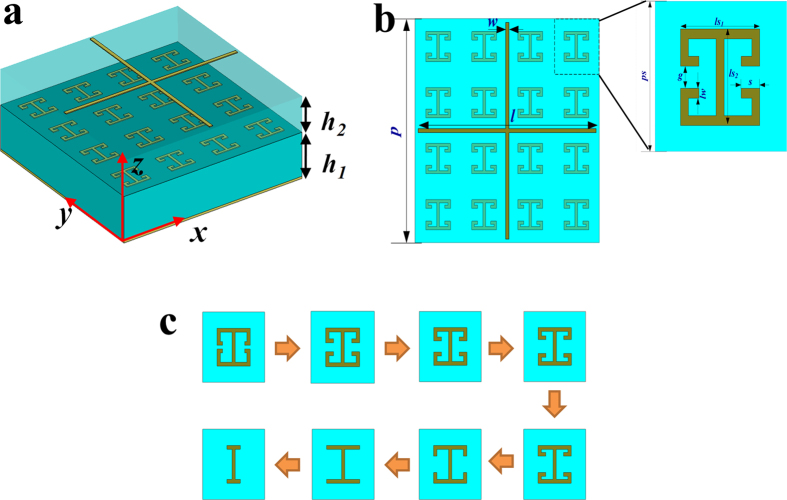
Schematic of unit cell for the proposed metasurface. (**a**) 3D-view of the unit cell used in the simulation. The metasurface is a combination of the I-shape structures and cross-line structures, and both two metallic patterns are printed on the same substrate with different thicknesses. The plane wave is normally incident onto the unit cell with electric field along *y* direction. (**b**) The front view of the unit cell. (**c**) Topological morphing route of the I-shape pattern.

**Figure 2:**
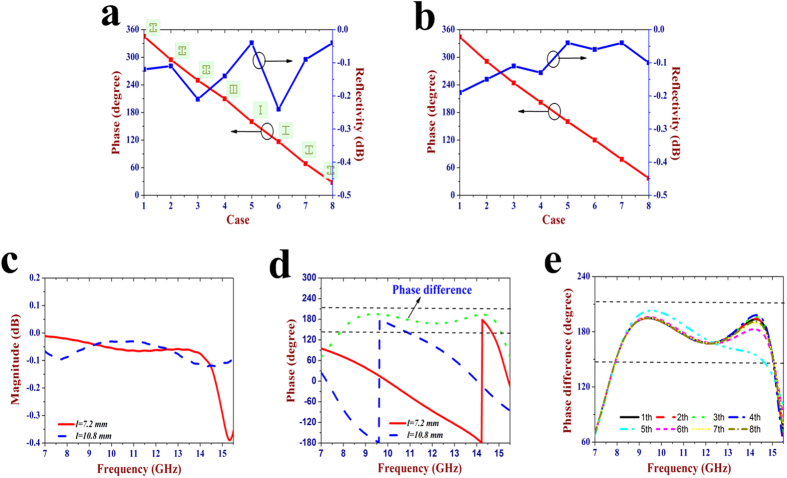
Full-wave simulation results of the unit cell for the proposed metasurface. (**a,b**) Simulation results of the bottom-layer metasurface at 25 GHz: (**a**) The reflection phase and magnitude for a series of I-shape patterns, in which the geometries of the eight I shape structures are given and all the calculation dates are obtained at the cross-line length of *l *= 7.2 mm. (**b**) The reflection phase and magnitude for a series of I-shape patterns at the cross-line length of *l *= 10.8 mm. (**c–e**) simulations results of the top-layer metasurface at 7–15.5 GHz: (**c**) The reflection magnitude of the cross-line structure with two different lengths, in which the calculation dates are obtained at the first I-shape pattern. (**d**) The reflection phase of the cross-line cell with two different lengths, which shows that the reflection phase difference is between 143° and 217° at 7.8–15 GHz. (**e**) The reflection phase difference at all the eight cases of the I-shape patterns.

Figure 1(a,b) present the schematic of unit cell for the proposed metasurface. It is composed of two layers metallic patterns. The bottom-layer metasurface utilizes the topological morphing of the I-shaped patterns which are etched on a grounded substrate with a thickness of 2 mm. The cross-line structure is adopted to construct the top-layer metasurface that is separated from the bottom-layer metasurface by an identical dielectric substrate with a thickness of 1.6 mm, as seen in Fig. 1(a). The dielectric constant of the selected substrate is 2.65 and its tangent loss is 0.001. The width of the cross-line structure is set to be *w *= 0.2 mm, while the initial value of its length is assumed to be *l *= 7.2 mm and then it is changed to tune the reflection phase. The I-shape structure is dependent of five geometrical parameters. Except for the value of *lw* and *ls*_*2*_, other three parameters, including *g*, *s,* and *ls*_*1*_, can be varied to obtain the large phase tuning range, as seen in Fig. 1(b). Figure 1(c) shows the topological morphing route of the I-shape pattern. By tuning its geometrical parameters, full reflection phase control with a range of 360° could be realized. The period of the top-layer metasurface is *p *= 11.2 mm, while it is *ps *= 2.8 mm for the bottom-layer metasurface. Both two layers of metasurface should be independently controlled to manipulate the reflection phase at different frequency bands. That is to say, there is no coupling effect between the top- and bottom-layer metasurface. In order to investigate the reflection characteristics of the designed metasurface, numerical simulation is carried out by using a commercial software CST microwave studio 2014. The whole metasurface is illuminated by a *y*-polarized wave, and periodic boundary conditions are set to its *x* and *y* sides. Figure 2(a) shows the reflection phase and magnitude of the bottom-layer metasurface at 25 GHz. Eight I-shape structures with different dimensions are designed to produce the gradient phase distribution. The sketches of each I-shape structure are depicted in the inset of Fig. 2(a). The values of *ls*_*2*_ and *lw* are kept fixed to be 1.5 mm and 0.15 mm for all the eight cases, while other three parameters are tuned as follows: *g *= 0.55 mm, 0.45 mm, 0.35 mm, 0.15 mm, 1.2 mm, 1.2 mm, 0.65 mm, 0.65 mm; *s *= 0.35 mm, 0.35 mm, 0.35 mm, 0.25 mm, 0.15 mm, 0.15 mm, 0.15 mm, 0.35 mm. *ls*_*1*_* *= 1.5 mm, 1.5 mm, 1.5 mm, 1.5 mm, 0.6 mm, 1.5 mm, 1.5 mm, 1.5 mm. The simulation results show that the varying parameters of the I-shape structures can effectively modulate its reflection phase, and the phase shift between neighboring I-shape structure cells is about 45°. In addition, the reflectivity of all the eight I-shape structures are larger than −0.3 dB, indicating that most of the incoming wave is reflected and then manipulated by the bottom-layer metasurface. The above simulation is obtained at the cross-line length of *l *= 7.2 mm. When the value of *l* is changed to be 10.8 mm, the eight I-shape structures still keep the gradient phase distribution with a phase step of about 45°, as seen in Fig. 2(b). Hence, we can conclude that the parameter variation of the top-layer metasurface almost has no influence on the reflection performance of the bottom-layer metasurface at 25 GHz. Figure 2(c,d) depict the reflection magnitude and phase of the top-layer metasurface, respectively. When the length of cross-line structure is, respectively, set to be 7.2 mm and 10.8 mm, these two meta-atoms can achieve almost full-reflection and their reflection losses are both less than 0.4 dB. The reflection phase difference is close to 180° at a broad band ranging from 7.8 GHz to 15 GHz. Through combining the two meta-atoms in a special arrangement, we can control the backward scattering field distribution to create diffusion effect so that the reflections of the normally incident waves could be effectively suppressed. Therefore, the top-layer metasurface is expected to achieve RCS reduction. Generally, 180° absolute phase difference is only restricted to a few frequencies. In the implement of the whole design, phase difference within 180° ± 37° can be accepted, which may result in 10 dB RCS reduction in theory^34^. Figure 2(e) shows the phase differences between the two meta-atoms under eight different I-shape structures. It is seen that the phase difference is still kept between 143° and 217° at 7.8 −14.6 GHz for all the cases. With the change of the parameters for I shape structures, the reflection phase difference almost has no obvious variation except for the fifth case where there is about 30° phase variation between 13 GHz and 14.5 GHz. Therefore, we have demonstrated that the top-layer metasurface and bottom-layer metasurface could be independently designed at two different frequency bands, which also paves the way for the design of a multi-functional metasurface.

**Figure 3:**
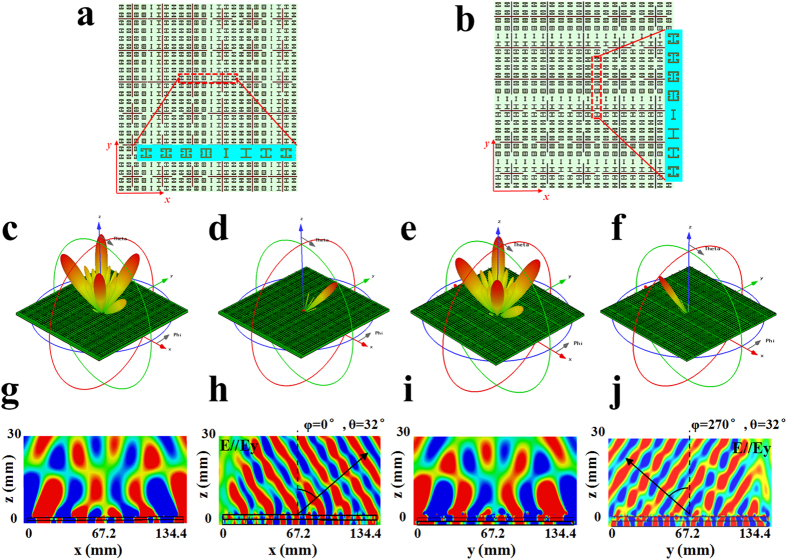
Full-wave simulations of the metasurface to verify its capability in the different functional control of the reflected wave at two frequency bands. (**a,c,d,g,h**) Schematic of the first metasurface and its simulation results: (**a**) Schematic of the metasurface, in which two kinds of cross-line structures are distributed in a chessboard-like configuration and eight kinds of I-shape structures are distributed with a certain phase shift along *x* direction. (**c**) 3D-scattering pattern at 11 GHz. (**d**) 3D-scattering pattern at 25 GHz. (**g**) Electric field distribution of the *y*-polarized reflection wave at 11 GHz in *xoz* plane. (**h**) Electric field distribution of the *y*-polarized reflection wave at 25 GHz in *xoz* plane, in which the reflection wave is deflected to (*φ *= 0°, *θ *= 32°). (**b,e,f,i,j**) Schematic of the second metasurface and its simulation results: (**b**) Schematic of the metasurface with eight kinds of I-shape cells distributed along *y* direction. (**e**) 3D-scattering pattern at 11 GHz. (**f**) 3D-scattering pattern at 25 GHz. (**i**) Electric field distribution of the *y*-polarized reflection wave at 11 GHz in *yoz* plane. (**j**) Electric field distribution of the *y*-polarized reflection wave deflected to (*φ *= 270°, *θ *= 32°) at 25 GHz in *yoz* plane.

Utilizing the proposed meta-atoms, we present the design of a multi-functional, multi-spectral metasurface. It is expected to create broadband diffusion at X-Ku band and meanwhile manipulate the incoming wave to the desired reflection direction at K-band. The schematics of the reflection-type metasurfaces with two different configurations are shown in Fig. 3(a,b), respectively. Each metasurface is composed of 12 × 12 top-layer cells and 48 × 48 bottom-layer cells. The design for these two metasurfaces is the same on the top layer where two kinds of 3 × 3 cross-line structures are combined in a chessboard-like configuration. The bottom layer on each metasurface is composed of one kind of combination of I-shape structures. In Fig. 3(a), eight kinds of I-shape structures with gradient phase distribution are arranged along *x* direction, while they are placed along *y* direction in Fig. 3(b). A *y*-polarized plane wave is incident onto the designed metasurface, and 3D scattering pattern at the frequency of interest can be obtained. It is seen in Fig. 3(c,e) that no matter which distribution is selected for the bottom-layer metasurface, the top-layer metasurface under normal incidence shows the obvious diffusion behavior, and its backward scattered wave is divided into four main beams at 11 GHz in the four quadrants, *φ *= 45°, 135°, 225° and 315°. Hence, the normal reflection of the incoming wave is sharply suppressed, leading to the obvious RCS reduction. In the higher frequency of 25 GHz, there is an anomalous reflection effect caused by the phase discontinuities in the bottom-layer metasurface. When a series of the I-shape structures are arranged along *x* direction, the reflected wave is deflected to (*φ *= 0°, *θ *= 32°), as seen in Fig. 3(d). If the arrangement of these I-shape structures is rotated by 90°, the deflection plane of the reflected wave is also changed to the other orthogonal plane, and its deflection angle is (*φ *= 270°, *θ *= 32°), as seen in Fig. 3(f). The corresponding electric field distributions on the observation planes vertical to the metasurface are shown in Fig. 3(g–j), respectively. They can further demonstrate that the proposed metasurface can create the diffusion effect by controlling the backward scattering into other directions at the lower frequency, and also simultaneously has the ability in deflecting the reflected wave to the given direction at the higher frequency.

**Figure 4:**
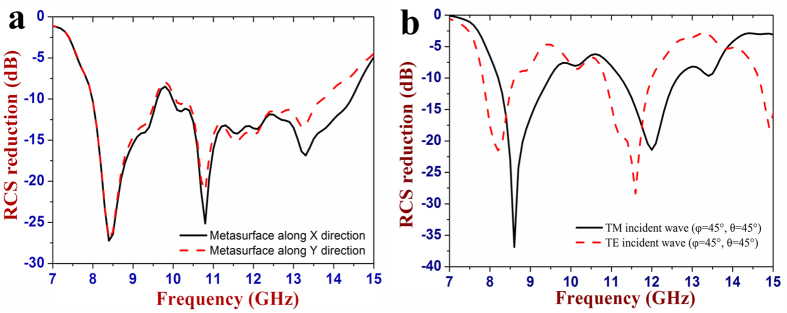
Simulated RCS reduction performance of the designed metasurface. (**a**) Normal incidence. (**b**) Oblique incidence with an incident angle of (*φ *= 45°, *θ *= 45°).

The RCS characteristic of the proposed metasurface (see Fig. 3(b)) is calculated over a wide frequency range from 7 GHz to 15 GHz, and then compared with that of a metallic flat plate with the same size. Figure 4(a) shows the RCS reduction result. It is seen that the proposed metasurface under normal incidence can significantly reduce the RCS at a broad band, and the bandwidth for 10 dB RCS reduction is about 5.7 GHz ranging from 7.8 GHz to 13.5 GHz. The maximum RCS reduction is as great as 26 dB. In addition, it is still seen that the RCS reduction performance is almost the same for the *x*- and *y*-polarized incoming wave, indicating insensitivity of the proposed metasurface to the polarization state of the normal incident wave. In addition, we also investigate the RCS reduction performance of this metasurface illuminated by a plane wave with an incident angle of (*φ *= 45°, *θ *= 45°), and its simulation result is given in Fig. 4(b). Compared with the situation of the normal incidence, the RCS reduction performance is obviously degraded, but the average RCS reduction value still reaches about 5 dB between 7.8 GHz and 13.5 GHz for both TE and TM polarizations.

### Experimental results

**Figure 5:**
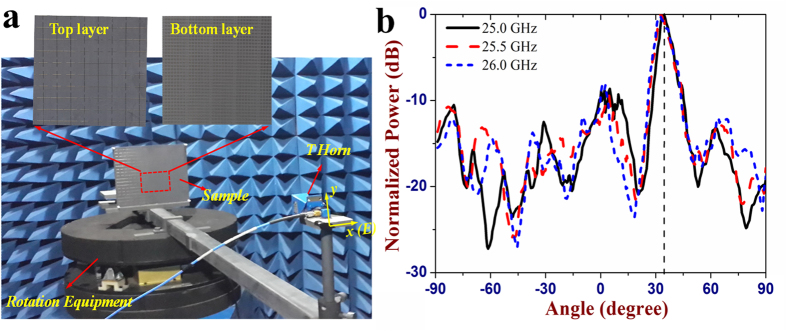
The beam deflection measurement of the fabricated metasurface. (**a**) Photograph of the measurement setup for the beam deflection. (**b**) The measured far field pattern of the metasurface.

**Figure 6:**
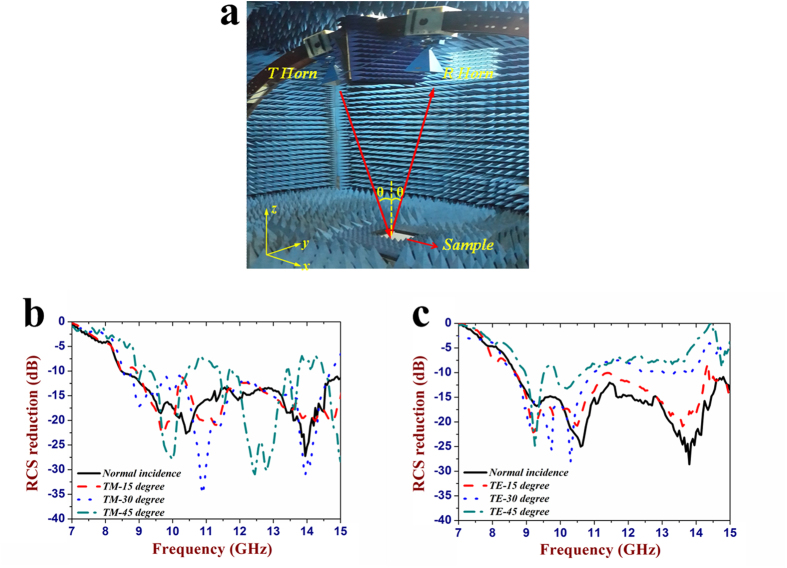
The RCS measurement of the fabricated metasurface. (**a**) Photograph of the RCS measurement setup. (**b,c**) The measured RCS reduction versus frequency from 7 GHz to 15 GHz at different oblique incidence angles: (**b**) TM polarization. (**c**) TE polarization.

In order to verify the simulation results of the designed metasurface, the second metasurface consisting of 18 × 18 unit cells has been fabricated by PCB technology and measured in the anechoic chamber. During the fabrication, the commercial dielectric board of F4B is selected for the substrate of this metasurface, which has the same parameters as those in the simulations. Figure 5(a) shows the measurement setup for the beam deflection. Two linearly-polarized (LP) K-band horn antennas connected to a vector network analyzer (R&S ZVA40) are utilized as transmitter and receiver, respectively. Both two horn antennas are horizontally polarized, which can excite the I-shape cells of the sample. The sample and the transmitting horn antenna placed in front of it are fixed on the rotation equipment. When moving the rotation equipment, the sample is always illuminated by the normal incident wave produced by the transmitting horn. The reflected wave for the (−90° to +90°) rotating angles can be received by the other horn antenna. The measured far field pattern is depicted in Fig. 5(b). It is seen that the normal incident beam is redirected to about 33° from 25 GHz to 26 GHz, violating the conventional Snell’s law. The measured beam deflection angle is in a good agreement with the theoretical simulation value of 32°. The RCS measurement setup is shown in Fig. 6(a). The two LP horn antennas are placed on an arch range and the sample is located in its centre. The incidence and reflection angles are both fixed at 5° to approximate the normal incidence condition. The distance between the antenna and sample is about 2.5 m, which is far enough to avoid the near field effect. In order to reduce measurement error, the sample is surrounded by the absorbing material, and the between two horns, the absorbing material is inserted for suppressing EM coupling. The RCS of this metasurface was measured over the frequency band of 7–15 GHz for both TE and TM polarizations. The corresponding results are compared with that of metallic flat plates with the same dimension, as seen in Fig. 6(b,c). For the normal incidence, the RCS of this metasurface is sharply reduced by 10 dB in 8.5–15 GHz under both TE and TM polarizations. Thus, the relative bandwidth for the 10 dB RCS reduction is calculated to be over 55%, showing excellent broadband property. The maximal RCS reduction is found to be as high as 25 dB around 14 GHz. In addition, the RCS reduction performance of the designed metasurface under different oblique incidence angles is discussed. With the increase of the oblique incidence angle, the RCS reduction is also considerable as expected, but its bandwidth for 10 dB RCS reduction is obviously decreased due to the phase aberrations. As we can see from the measured results, the RCS is reduced by about 6 dB between 8.5 GHz and 13.5 GHz for both TE and TM polarizations with each incident angle. That means the designed metasurface still has the ability in modulating the backward scattered waves under oblique incidence with large angles.

## Discussion

In summary, we have proposed a multi-spectral metasurface that has different functions at each frequency band. This metasurface is composed of two layers metallic patterns, and each metallic pattern can independently manipulate the reflected field distribution at the corresponding frequency through special design. Both simulated and measured results demonstrate that the designed metasurface can deflect the incoming wave to the given direction at 25–26 GHz, and simultaneously achieve obvious RCS reduction by producing diffusion effect at a wide frequency band ranging from 8.5 GHz to 15 GHz. Compared with single function of the previous metasurface^36^, the designed metasurface not only integrates stealth technology, but also has detecting function if integrated with a horn source to construct the reflectarray antenna^37^. This work provides a new route for the multi-functional control of EM wave, which could be developed for potential stealth applications in the future.

## Additional Information

**How to cite this article**: Huang, C. *et al.* Multi-spectral Metasurface for Different Functional Control of Reflection Waves. *Sci. Rep.* **6**, 23291; doi: 10.1038/srep23291 (2016).
